# The Use of POSS-Based Nanoadditives for Cable-Grade PVC: Effects on Its Thermal Stability

**DOI:** 10.3390/polym11071105

**Published:** 2019-06-29

**Authors:** Luca Palin, Giuseppe Rombolà, Marco Milanesio, Enrico Boccaleri

**Affiliations:** 1 Dipartimento di Scienze ed Innovazione Tecnologica (DiSIT), Università del Piemonte Orientale, Viale T. Michel, 11, 15121 Alessandria (I), Italy; 2 Nova Res S.r.l., Via D. Bello, 3, 28100 Novara (I), Italy

**Keywords:** plasticized poly(vinyl chloride), PVC, cable application, nanocomposites, nanomaterials, thermal stability extrusion, POSS, hydrotalcites, zeolite X, HCl scavenging

## Abstract

Plasticized–Poly(vinyl chloride) (P-PVC) for cables and insulation requires performances related to outdoor, indoor and submarine contexts and reduction of noxious release of HCl-containing fumes in case of thermal degradation or fire. Introducing suitable nanomaterials in polymer-based nanocomposites can be an answer to this clue. In this work, an industry-compliant cable-grade P-PVC formulation was added with nanostructured materials belonging to the family of Polyhedral Oligomeric Silsesquioxane (POSS). The effects of the nanomaterials, alone and in synergy with HCl scavenging agents as zeolites and hydrotalcites, on the thermal stability and HCl evolution of P-PVC were deeply investigated by thermogravimetric analysis and reference ASTM methods. Moreover, hardness and mechanical properties were studied in order to highlight the effects of these additives in the perspective of final industrial uses. The data demonstrated relevant improvements in the thermal stability of the samples added with nanomaterials, already with concentrations of POSS down to 0.31 phr and interesting additive effects of POSS with zeolites and hydrotalcites for HCl release reduction without losing mechanical performances.

## 1. Introduction

Polyvinylchloride (PVC) is a polymeric material, employed in a huge number of applications, ranging from medical devices, automotive, flooring, luxury goods, clothing and construction [1]. The benefits of PVC as a commodity polymer rely in its good performances in a wide range of temperatures (from −40 °C to 125 °C), in the resistance to environmental ageing (i.e., UV light stability) and chemicals as oil and gasoline and in the compatibility with biological fluids and transparency. Under the technological point of view, PVC advantages are related to the ease of processing, ranging from blending to moulding and extrusion, and the versatility of its possible formulations, resulting in materials with rigid (unplasicized–U-PVC) and flexible (plasticized–P-PVC) behaviors. Concerning construction and building applications, PVC uses are featured by particularly long lifetimes (from 10 to 100 years) with respect to other thermoplastics, employed i.e., for water and sewage piping, for windows frames and for the insulation of electric cables or for flooring. These applications exploit over 60% of Western Europe’s annual PVC production [2]. For electric and electronic applications specifically, P-PVC cables account for 46% of the whole European cable market in 2016, with a share in the low-voltage cables sector reaching 70% [3]. Cable-grade P-PVC is used for sheathing and insulation of electrical and data transmission cables for domestic, commercial and industrial electric power distribution infrastructures, conventional and electric/hybrid automotive wiring and industrial robotics. For these fields, aside from the inherently high value of the electrical insulation coefficient, key features are the self-extinguishing capability and the fire resistance. PVC is difficult to ignite, does not sustain combustion nor does it contribute to flame propagation. Moreover, under heating, PVC does not generate flaming droplets and produces a low amount of smoke. Finally, when considering the whole life cycle, most of the P-PVC cables is currently reused and recycled, with a consequent reduction of its footprint and impact [2]. 

To match the expected performances, P-PVC products are currently formulated using a large number of additives as stabilisers and lubricants operating during the production process, plasticisers, processing aids, impact modifiers and pigments. The fundamentals on standard P-PVC additives and formulations are widely detailed in the literature [4]. Low-cost physical fillers such as calcium carbonate or other inorganics are traditionally prevalent, used in amounts up to 60–80 wt.%, mainly to balance a reduction of amount of resin with acceptable mechanical features and a sustainable price of the final formulation. Since the end of the 1990s, last century polymer compounding was disruptively changed by the emergency of the polymer-based nanocomposites science [5], with the use of nanostructured additives able to reach a dispersion at submicrometric scale. While several matrices have been thoroughly investigated in scientific publications, a systematic work [6,7,8] based on polyvinylchloride (PVC), also with the purpose of providing knowledge and guidelines for industrial applications, is still lacking. This paper aims at filling this gap working on flexible cable-grade P-PVC formulations, focusing specifically on the main concerns of this applicative field. 

The principal drawback for P-PVC formulations for electric and electronic application is the production of aggressive HCl-containing fumes in case of thermal degradation and combustion, and the main challenge is further improving the heat resistance, the thermal stability upon time and the reduction of noxious and toxic emission in case of heating and fire. Heating promotes the elimination of HCl from the PVC backbone that in turns self-accelerates the degradation with the so-called unzipping mechanism. The onset of dehydrochlorination unlocks an avalanche effect, with release of HCl fumes and the formation of distributed unsaturations within the polymer, causing the loss of the chemical and physical features and the change of colour to brown/black [9]. Thermal stabilisation of PVC can be operated by the so-called primary stabilisers, that reduce or hinder the unzipping reaction, or secondary stabilisers, that work on scavenging HCl or Cl∙radicals avoiding the chain propagation reaction [6]. The role of stabilisers was related to two main parameters: the induction time (i.e., the time lapse at a certain temperature before the HCl evolution starts taking place) and the rate (or the temperature) where the dehydrochlorination occurs massively [10].

P-PVC formulations for cables, however, need to side the amount and rate of HCl evolution upon heating and the resistance to ageing to the preservation or improvement of rheology, mechanical resistance and flexibility during processing and use. Furthermore, additives should also be preferably colourless, odourless, compatible to the polymeric matrix, non-migrating and cost effective [6]. In this work, a series of nanostructured materials specifically designed for plasticised P-PVC and matching several of the desirable features [4,6] was investigated, using innovative hybrid inorganic-organic 3D nanomaterials known as polyhedral oligomeric silsesquioxanes (POSS) [7,11]. POSS additives were used alone at first and then in synergy with other functional additives such as HCl scavengers, to improve thermal properties of P-PVC while preserving workability and mechanical properties. In particular, the studied formulations included POSS in combination with commercial X-type zeolite (X-ZEO) [12] and carbonate-substituted hydrotalcite (HTLC [13]). HTLC was chosen because of the reported effectiveness on PVC stabilization [10,14,15,16,17]. Its role (in general referred to its carbonate form) is claimed to be related both to the reactivity and exchangeability of the interlayer anions [18,19,20,21,22,23,24,25,26,27,28,29,30], but also to their acid-base surface reactivity [16] and the electrostatic interactions between the positive layers of this hydroxide and the polymer chains, that weaken the ability of chlorine atoms to undergo dehydrochlorination [10,21]. Regarding X-ZEO, the choice was driven by its capability to entrap HCl in its porous structure [22,23]. As a keystone to meet the industrial interests, both P-PVC formulation and nanoadditives were chosen to be close to commercial applications, with wide availability and sustainable industrial cost. A P-PVC mix design composed of basically all the conventional co-formulants required for an industrial extrusion process was employed, and among the world of nanoadditives, a cutoff selection was targeted to use materials that can be purchased in tons scale on the market. Previous studies concerning the use of POSS in PVC matrices were carried out concentrating on a specific POSS and not in combination with porous or layered materials [24,25,26].

Processing of the polymers, made by dry blending, compounding and extrusion were performed on the scale of about tenth kilograms using pilot scale machinery, fully compliant with the industrial specifications for production, to better meet the interests of compounders. Finally, the produced materials were characterized by fundamental lab methods as Thermogravimetrical Analysis and compared with industrial reference tests (ISO/ASTM/CEI EN standards) for cable-grade formulations characterization, to understand how the molecular features of the nanoadditives, their amount and their nanometric dispersion can modify the P-PVC properties to better exploit the effects due to nanosized additives and reaching relevant final performances.

## 2. Materials and Methods 

### 2.1. Materials

The reference P-PVC (REF) for cable applications is a formulation containing a standard series of co-additives in order to tailor the properties for specific process conditions and final features. A typical industrial formulation for cable-grade P-PVC was selected, whose composition (according to the common formulation procedure based on p.h.r.–parts for hundred resin) is reported in Table 1 [27].

**Figure 1:**
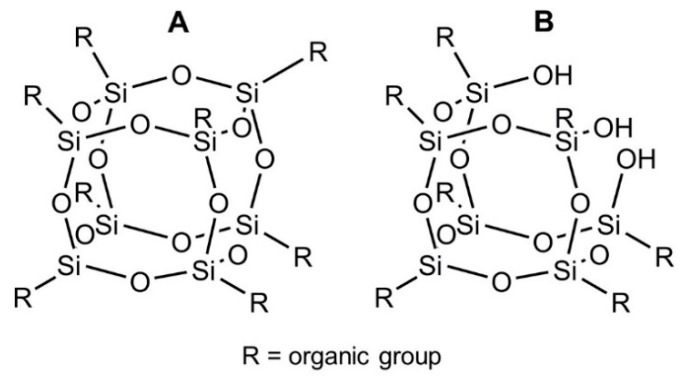
Completely (**A**) and partially condensed (**B**) polyhedral oligomeric silsesquioxanes (POSS).

POSS were purchased from Hybrid Plastics Inc. (Hattiesburg, MS, USA) and used as received. POSS were selected on the basis of the technical data sheets provided by the producer. POSS basically can be featured by a closed cage (Figure 1A) with a cubic Si-O structure with different organic pendant groups, o by an open cage (Figure 1B), with seven R pending organic groups and three Si-OH units.

In this work, close cage POSS were used with Vinyl functionality (VyPOSS, produced as a cage mixture with 8 and > 8 corners – commercially OL1170) and glycidyl propyl ether groups (GlyPOSS, produced as a cage mixture with 8 and > 8 corners–commercially EP0409). Open cage POSS were employed with isobutyl (IBuPOSSOH–commercially SO1450) and phenyl (PhPOSSOH–commercially SO1458) organic moieties^11^.

X-zeolite was a Zeolum F9 X-type Zeolite Na^+^ exchanged, in powder form with 100 mesh granulometry, purchased from Tosoh Inc. and used as received^12^. 

Hydrotalcite was purchased by Sasol as commercial sample PURAL® MG 61 HT, with a layer composition based on MgAl hydroxides (Al_2_O_3_:MgO 39:61 wt.) and carbonate ions (10 wt.%) as interlayer anions^13^. 

This reference resin formulation was added with POSS and other functional fillers firstly alone to assess the best performing materials and explore the effects of concentration on thermal performances and workability. Secondly, the best performing materials of Table 2 were used in combination with X-Zeolite and HTLC as detailed in Table 3. The REF sample, without any nanofiller, and thus containing only standard additives of Table 1 was processed and formed in the same way as the samples in Table 2 and Table 3. 

### 2.2. P-PVC Ribbon Preparation

Powdered P-PVC and nanoadditive(s) were treated with conventional industrial dry blending apparatus for 2 minutes using a mixing profile varying the mixing speed from 1000 to 2900 rpm [28] and then extruded to pellets on a Maris TM 20 HT – twin screw co-rotating extruder, operated with a feed rate of 7 kg/h, with a l/d ratio of 40, D/d ratio of 1.5, screw profile of 3.5 mm. The temperature along the extrusion cylinder (over 13 temperature zones) were respectively set as 30, 100, 100, 100, 100, 100, 100,160, 160, 160, 140, 140, and 160 °C. The output, in the form of 4 mm pellets were air cooled and collected. Pellets were then processed in a conventional single screw extruder with a shaped drawing to provide 40 × 4 mm ribbons, followed by a roll calendrer and air cooling.

### 2.3. Characterization Methods

The P-PVC ribbons were studied according to standard methods listed in Table 4. All the measurements according to standard methods were carried on in certified laboratory structures.

Thermogravimetrical analysis (TGA) was performed on a SetSys Evolution instrument by Setaram (Caluire FRANCE). As reported, samples were measured in ramp heating conditions (RT–800 °C) with a heating rate of 10°C/min and under an Ar flow of 20 mL/min. Isothemal measurements were performed with the same apparatus, setting the temperature using a fast heating to target temperature (>30 °C/min), using Ar flow at 20 mL/min.

## 3. Results

The effects of nanoadditives in Table 2 were firstly evaluated during the processing, by monitoring the torque values of the twin screw extruder, to highlight the effect on rheology and to tune the process parameters. Once the amounts of additive were defined in order to afford reproducible and reliable processing, the thermal stability was investigated using ramp and isothermal TGA measurements providing complementary information as detailed in the results and discussion section. Complementary information on HCl evolution and mechanical properties were obtained by ISO/ASTM/CEI EN measurements for thermal stability, Limiting Oxygen Index (LOI) and mechanical properties.

**Figure 2:**
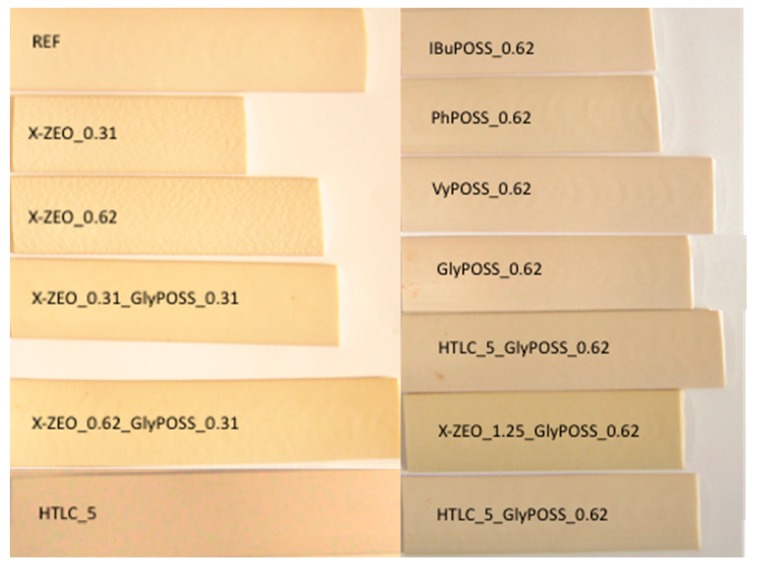
Picture of nanocomposite P-PVC formulation ribbons.

The most effective additives were then used in combination, coupling hybrid (i.e., POSS) with inorganic fillers (HTLC or X-ZEO) to explore the effects of their co-presence. P-PVC nanocomposite samples under the form of ribbons are shown in Figure 2.

### 3.1. Addition of POSS: Effects on Rheology

POSS nanoadditives show remarkable effects already during the extrusion process. POSS, during the melt processing in the twin screw extruder, change relevantly the viscosity of P-PVC due to their homogeneous distribution at nanoscale level. The process torque value recorded during extrusion of the formulations is reported in the ESI section (Table S1). In a preliminary test, with the extruder working at 90 rpm with a high loading (1.25 phr) of Isobutyl POSS trisilanol (IBuPOSSOH_1.25), a dramatic change in the motor torque was recorded due to a relevant drop in viscosity. This result, already observed for the presence of POSS in other matrices [29], highlights that the dispersion efficacy has reached the molecular level and that POSS perturbates heavily the interactions of PVC chains. The final extrusion conditions, balancing at best the dispersion of fillers, were then achieved setting the extruder at 150 rpm and modifying the POSS addition. Reduced amounts of POSS (0.62 phr) provide torque values similar to the REF sample but can prevent dramatic increases of viscosity when coupled with other fillers (i.e., with X-ZEO at 0.31 phr), promoting their dispersion as well.

On the basis of the process parameters, only the set of samples with one and two additives providing flow features similar to the REF sample were further characterized from the thermal stability viewpoint, while the high flow formulations were discarded because they are scarcely relevant in real applications, being less or not workable in an industrial plant.

### 3.2. Thermal Stability Analysis by TGA Measurements 

For the complete understanding of the results of TGA analyses, a premise on PVC degradation steps is mandatory. In the degradation of PVC, the release of HCl causes an autocatalytic effect promoting a first step of generation of conjugated double bonds along the polymer chain, and a second step of condensation, where Diels Alder cyclisation and cross-linking of polyene segments cause the formation of aromatic fractions [30], and the gradual darkening of the polymer [31]. This behavior is related, in the literature, to different factors promoting the degradation, as the occurrence of tertiary chlorine containing species that play as “labile sites”, allylic chlorine species siding a double bond occasionally present [6], isotactic anomalies or in general defects due also to the processing, oxidation or ageing. Detailed kinetic studies highlight that the dehydrochlorination process (expected to occur up to 350 °C) is characterized by two steps with different mechanisms: the first degradation (occurring at lower temperature) is featured by a nucleation and growth model, with degradation promoted by discrete points (i.e., particularly labile sites, defects, irregularities) progressing then in the rest of the material. The second dehydrochlorination step is instead diffusion controlled, giving rise to bubbles of HCl within the residual polymer structure, and sensitive, in the HCl evolution, to the degree of cross-linking of the degraded polymeric fraction [32]. 

Samples were tested using TGA under inert (Ar flow) condition both in temperature ramp and in isothermal conditions. In ramp heating measurements, the calculation and graphical representation of the DTG derivative (differential mass loss- wt.%/°C) highlights the presence of the two degradation steps described above. Using both TGA and DTG profiles, the effects of the nanofillers on the degradation mechanism were evaluated in terms of different parameters. From TGA plots, the increase of the thermal stability was related to the shift in temperature of the onset of degradation. The onset point was conventionally defined as the temperature where the degradation rate reaches the 0.5 wt.%/°C and this temperature was established on the basis of the behaviour of REF sample. The temperature shift (and also a plot shape changes) for the maximum rate of HCl evolution after the onset (highlighted by the DTG plot) was related to the stabilization of the primary dehydrochlorination, operated on the nucleation process by removing or avoiding the degradation of labile sites. Afterwards, at higher temperature, changes in the DTG profiles were related to the impact of nanofiller(s) on secondary processes (secondary dehydrochlorination and conjugation) due to stabilization or hindering its evolution or absorbing it without release in the gas phase.

**Figure 3:**
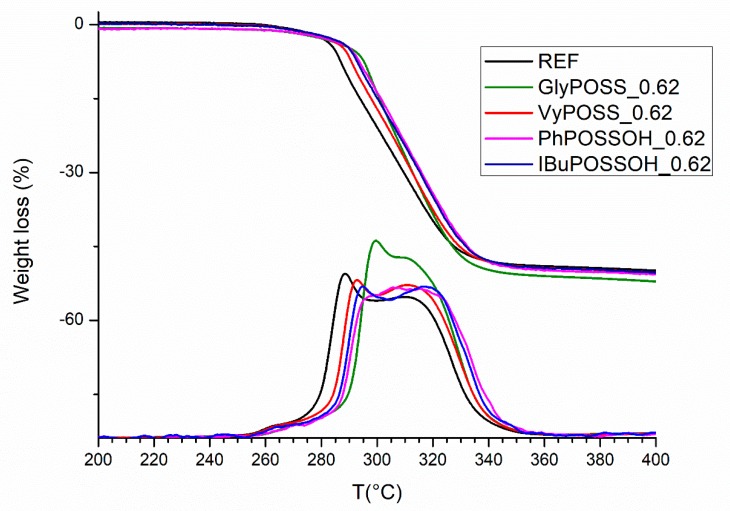
TGA/DTG analyses of reference P-PVC (REF, black) and POSS formulated P-PVC according to Table 2 formulations.

The ramp TGA profiles of samples obtained with a single additive are reported in Figure 3. The thermal profile of REF highlights the onset of the decomposition process, that, after a preliminary weight loss starting at 250° C (probably due to standard additives in the formulation listed in Table 1) begins at 258 °C and reach the stated onset conditions at 265 °C, a subsequent thermal degradation process reaching the maximum rate of decomposition (i.e., the maximum of the DTG curve) around 288 °C, and a second degradation process, with a peak rate at 311 °C rate and distributed in temperature up to 350 °C, with a loss of about 50% of weight of the polymer. When POSS are added, the effects on the thermal behavior are due to relevantly low amounts (0.6 phr) of POSS differing in the chemical features (i.e., close vs. open cage, different R pendant groups). P-PVC containing close cage Vinyl POSS shows a delay of the thermal processes (+1 °C for the onset), with a shift of both the first degradation path and second decomposition process (from the maxima in DTG profile) of about +7 °C. The use of open cage POSS with different R groups (Isobutyl or Phenyl) highlights some further difference in behavior. Phenyl POSS causes the reduction of the decomposition rate of the first process, that appears mixed with the second degradation effect, while glycidyl substituted POSS shifts relevantly the first degradation of about +11 °C with a remarkable effect on the thermal stability of the formulation.

These evidences underline an active role for POSS with potentially reactive R groups (i.e., OH on silanols, vinyl and glycidyl pendant groups); despite the low concentration, their homogeneous distribution in the polymer favour the interactions with the P-PVC matrix that can take place either during the extrusion process or upon the thermal treatment. In particular, POSS presence seems to reduce the amount and/or the reactivity of the already claimed labile sites that are responsible of the preliminary formation of degradation “nucleation centers” [29]. 

The glicidyl POSS/inorganic nanomaterials synergy was preliminarily investigated in combination with X-ZEO to assess the best range of nanoaddition. Ramp TGA analyses on P-PVC formulated with amounts of X-Zeolite from 0.31 to 0.62 phr (Figure S1, left) highlight the role of HCl physical scavenger with a temperature shift of +3–+8 °C on the onset of thermal induced processes. A significant supplementary effect can be seen by the coupling of zeolite with glycidyl POSS at 0.62 phr, as a shift of temperature of +10 °C for the onset, +17 °C and +10 °C for the two degradation steps was found. It is noteworthy that glycidyl POSS, for its chemical composition, has no direct capability to perform HCl storage effect, though epoxidized oils are considered secondary stabilizers used to enhance the effectiveness of conventional additives [33], but in this case it clearly promotes and enhances the effect due to the presence of the zeolite. Further insights can be provided by TGA isothermal experiments. These measurements are interesting for the mechanistic information they can provide; in fact, if temperature is set at the onset of the degradation process (as said at the temperature where the degradation rate reaches 0.5 wt.%/°C for unadded REF formulation), it can allow the discrimination of a physical effect due to the presence of additives from a chemically active filler. This second, in fact, changes the pathway of degradation reactions towards final different products with a different profile in time and an increased residual mass with respect to the REF sample [34]. Conversely, if the role of the additive is basically physical, a reduction of the rate of the degradation process gives just a kinetic effect, with a slower convergence to the same residual mass of the REF sample.

According to the definition of onset temperature for REF P-PVC, isothermal TGA analyses were performed at 265 °C under Ar atmosphere. The study on GlyPOSS/X-ZEO formulations (see Figure S1 right) show that the decomposition in isothermal conditions is relevantly delayed by the presence of the zeolite, mainly within the first 1500 seconds but with differences in stability of the residue up to the end of the experiment. On the contrary, GlyPOSS alone has only some stabilization role in the 0–1000 seconds range. The combination of the two additives has evident gains depending on the zeolite concentration, with a distributed stabilization effect over the whole time of the experiment. 

**Figure 4:**
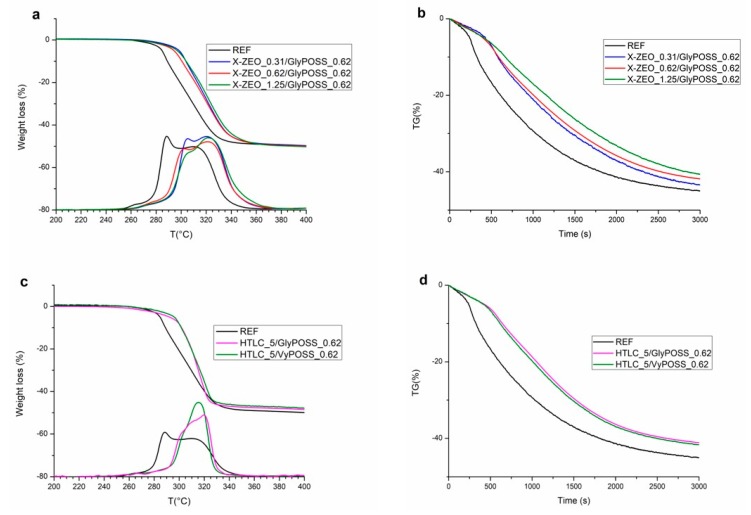
TGA analyses of (**a,b**) REF and X-type zeolite/POSS (0.31–1.25 phr); (**c,d**) P-PVC and of REF and HTLC/POSS P-PVC formulations in the compositional range 0.62–5 phr in ramp (**a**,**c**) and isothermal conditions (**b**,**d**).

This indication suggested an extensive investigation of inorganic nanomaterials in combination with GlyPOSS in concentrations around 1 phr. Figure 4, top shows on the left ramp TGA experiments run with the combination of zeolite X and glycidyl POSS with increasing concentration of zeolite from 0.31 to 1.25 phr. Notably, the effects of stabilization (i.e., the shift of the onset temperature) are consistent for all three formulations, while the rate and the relative rate of the first vs. second degradation path are influenced by the concentration of X-type zeolite. This behaviour, confirming the role of zeolites as HCl retainers [22] when the degradation is diffusion controlled [29], is emphasized by the isothermal experiments reported on the top right in Figure 3. All the composite formulations gain stability versus neat P-PVC over the first 600 seconds and after this time the composition of the three added samples differentiates them, with an increase of the stability along time and at the end of the experiment (after 3000 seconds) related to the increase of the amount of zeolite.

The combination of POSS with layered inorganic nanofillers, in particular a commercial carbonate-substituted hydrotalcite, is still more relevant (bottom in Figure 4). When layered materials are involved in thermal degradation of polymeric matrices, some concurrent effects can be present. In particular, a physical role for the presence of inorganic platelets, driven to the surface due to the ablation of the polymer upon degradation, can side the possible chemical role in promoting different chemical path of degradation [6] by a massive HCl adsorption [18]. The TGA plots (both in ramp heating and isothermal conditions at 265 °C) show that the presence of glycidyl and vinyl POSS (always at 0.62 phr) together with 5 phr of HTLC have a dramatic capability of modifying the thermal stability and degradation. As evident in DTG profile, with both POSS but in particular when vinyl POSS is present, the mechanism is no longer featured by two steps but a single, delayed one. This evidence is confirmed by the isothermal measurements—HTLC containing formulations, with the presence of POSS, have a relevant gain in thermal stability and in residual mass amount.

### 3.3. Overall Discussion on Thermal Stabilisation Effects of Nanoadditives

**Figure 5:**
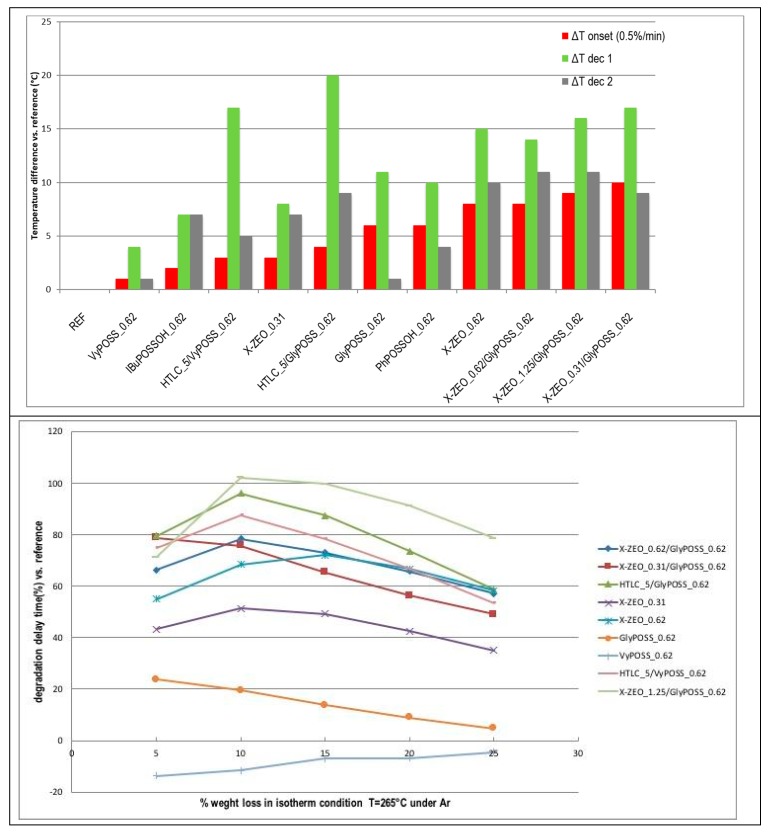
Graphical representation of temperature shifts (°C) of the degradation onset, the first and second decomposition process derived from ramp TGA (top) and degradation time shift derived from isothermal TGA (bottom) for nanocomposite P-PVC formulations vs. REF.

The effects of stabilization of the onset of degradation, shift of decomposition temperature for step 1 and 2 of degradation of P-PVC, as well as the effects of stabilization in isothermal conditions for several modified P-PVC formulations are summarised in Figure 5, top to highlight the onset temperatures and degradation delay in nanocomposite samples with respect to REF. The use of different nanoadditives and/or their combination can modify the whole decomposition behavior of P-PVC changing relevantly the onset of thermal degradation (red bar), the maximum rate of decomposition for primary dehydrochlorination (green bar) or the maximum rate of degradation for secondary dehydrochlorination process (grey bar). Chemical effects within the degradation process are evident from the graph in Figure 5, bottom, where the weight loss and the delay time with respect to the REF during isothermal experiments at 265 °C are compared. The plot shows well that samples containing only POSS may have relevantly different behavior; for instance, vinyl POSS at 0.62 phr reduces the isothermal stability vs. the REF, while glycidyl POSS at the same concentration provide a preliminary stabilization that vanishes during time with a straight linear trend. When P-PVC is added with zeolite or HTLC HCl scavengers, an evident effect of protection is detected, causing an increasing delay of decomposition. However, the combination of these HCl retainers and glycidyl and vinyl POSS make the isothermal stability even more effective. The convex profile of this curve suggests that the efficacy of the additives increases during the first part of the experiment.

### 3.4. Thermal Performances using ISO/ASTM Methods

To confirm the results given by TGA measurements, the standard method UNI EN ISO 182-3:2003 part 3 [35] was adopted to study the nano-added P-PVC in view of real-world technological applications. This test evaluates the time occurring before the beginning of release of HCl fumes upon heating at 180 °C (induction time) and performs also the measurement after a preliminary thermal ageing (residual thermal stability after 168 hours at 80 °C) that often makes the testing methodology very demanding for the materials properties. Results are reported in Table 5.

The REF sample has an induction time of 40 minutes, and the addition of POSS alone changes these results in the range between 45 and 60 minutes (i.e., from 12.5 to 50% of increase). The case of GlyPOSS_0.62 is interesting as there is a residual evident effect also after thermal ageing, where the induction time (though reduced to 87.5% of the pristine value) can be still observed. When POSS are coupled with zeolite or HTLC, formulations containing X-type zeolite and POSS gain between 20–25% of induction time increase, and formulations with POSS and HTLC reach the best performances both in direct test and after thermal ageing, though the role and effect of POSS is not evident.

Limiting Oxygen Index (LOI) measured according to ASTM D 2863 (see Table 5) on the samples further underline the efficacy of POSS formulations, in particular for Z_ZEO_0.31, PhPOSSOH_0.62 and GlyPOSS_0.62, hence used alone in the formulation. For these samples, though the best performance was reached by HTLC_5 (hydrotalcite alone at 5 phr addition) with a result of 26%, the results of these formulations (all at 25.5% of LOI) reveal the high efficiency of the co-formulated materials as this result was obtained with amounts of fillers around 1/10–1/20 of that of HTLC. These data confirm that the stabilization suggested by TGA are valid also within the stringent requests of an ISO/ASTM method.

The best performing sample (HTLC_5/GlyPOSS_0.62) according to previous analyses was also tested, together with samples with the two nanomaterials alone, with reference method for HCl evolution as CEI EN 50267-2-1/IEC 60754-1 to better highlight the role of POSS with HCl scavengers as HTLC (Table 6). The method is based on thermal heating of the sample up to 800 °C for 30 minutes and on the collection of HCl released by the sample. The presence of 5 phr of HTLC or 0.6 phr of glycidyl POSS alone respectively give a reduction of HCl evolution of 10.7 and 7.1% with respect to the REF sample. When they are combined, HCl amount released smaller than the sum of the two nanomaterials alone, but with a remarkable reduction with respect to REF of 14.3%.

### 3.5. Mechanical Properties

Physical properties like hardness are in general modified by the presence of nanoadditives; in several cases the materials are softened by the presence of the POSS, whether alone or in co-presence of dispersed X-type zeolite. When layered materials are present, an increase of hardness was found. The obtained good performances in thermal stability must be sided by mechanical properties not different from REF sample and the nanomaterials-containing formulations must be compliant to the requirements for potential industrial end-user applications. Mechanical tensile test ASTM D638 on standard dogbone samples (obtained by cutting the ribbons), and Sh.A (15’’) hardness test according to ISO 868 were thus carried out and the results are collected in Table S2 in the ESI file. As a first consideration, all the values fall within two or three times the st.dev with respect to REF sample. For instance, the elongation at break is often similar to the REF sample. This property is often dramatically influenced by the presence of additives, and in formulation based on two additives (i.e., X-ZEO_0.31/GlyPOSS_0.62) the presence of POSS completely counterbalance the reduced elongation effect of the addition of the X-zeolite and provide acceptable elongation at break even for formulations with 5 phr of HTLC. This ensures that nanomaterials do not affect dramatically the mechanical properties. It must be noted that some improvements are also observed. In the case of HTLC_5/GlyPOSS_0.62, the co-presence of GlyPOSS leads to a hardness value identical to the REF sample. Regarding tensile mechanical properties, all the formulations tested show the elastic modulus and stress at break basically unchanged.

## 4. Discussion

At first, POSS with different chemical features were molecularly distributed as unique nanoadditive within P-PVC proving their ability to promote a relevantly different thermal behavior for the unzipping reactions of P-PVC towards conjugated polyaromatic systems, and their efficacy was demonstrated with amounts between 0.3 to 1 phr.

The evidence of effects down to 0.3 phr concentration is probably related to the molecular nature of this nanomaterial, with organic moieties directly bound to silica cage. Its compositional and structural homogeneity can allow an improved distribution, reaching molecular level, with respect to other nanofillers. Once R groups are able to interact with the polymer, the distribution promote a widespread interconnection with the polymer. As a general consideration, it appears clear that POSS role in this study is significantly influenced by the nature of the cage structure (i.e., open vs. close cage structure) and the potential role of R modifiers (i.e., inert, as iBu- or Ph- vs. reactive groups as vinyl or glycidyl-). In all these cases, peculiar effects were seen on the thermal degradation under nitrogen. Among all the POSS, the most interesting results, balancing overall results about the thermal behaviour under the different test methodologies and the mechanical features, came from GlyPOSS. This material introduces a ceramic cage surrounded by epoxy terminated organic arms. The explanation of its relevant efficacy can be related to the role, known in literature, of epoxidized oils as secondary stabilizers used to enhance the effectiveness of conventional additives as metal soaps, as acceptors for the free hydrogen chloride [36,37] and retardants for the change in colour [38] coupled with efficiently distributed thermally stable ceramic nanoparticles.

Then, the use of POSS together with active inorganic materials able to interact with evolved HCl (X type zeolite and carbonate-substituted HTLC) proved that in the presence of amounts of inorganics between 1.25 and 5 phr, the decomposition profile is dramatically modified, and additives have a role in the different steps of thermal degradation of PVC that can be highlighted.

In particular, POSS and their combination with HTLC and X-ZEO highlight that the influence on the degradation steps of PVC provide a longer time stability in isothermal conditions. This effect is further enhanced with the evolution of the degradation for some formulations, as X-ZEO1.25/GlyPOSS0.62, HTLC5/GlyPOSS0.62 and HTLC5/VyPOSS0.62, which show up to 100% increase of delay time vs. reference in isothermal conditions (Figure 5, bottom). The evidences provided by TGA information were consistently confirmed by the measures of thermal stability and HCl release using standard reference methods. The improvement of the thermal stability, sided by the preservation of features like the processability, mechanical features and good performance under thermal ageing makes the use of POSS-based nanoadditives promising in widening the applications of P-PVC formulations, both in the field of cables where stability in temperature and HCl are critical issues, and for contexts where resistance to temperature is required during the production process. The most interesting combination of nanomaterials, balancing the improvement of thermal stability with reduction of HCl fumes with good mechanical features came from GlyPOSS in combination with zeolite X or hydrotalcite. 

## 5. Conclusions

Cable grade formulations based on P-PVC can be improved for their performances, in particular concerning thermal stability and HCl release reduction, by the use of nanomaterials in amounts relevantly smaller (down to 0.31 phr) than usually employed fillers for polymer compounding. HTLC_5/GlyPOSS_0.62 resulted the best performing sample when considering thermal stability and HCl release while maintaining mechanical properties similar to REF sample. It must be noted that POSS, POSS/Zeolite and POSS/HTLC systems have several beneficial aspects being colourless, odourless, tasteless and non-migrating [6]. The efficacy and the added amount (around 0.3 to 0.6 phr for the more expensive POSS fraction) can balance the cost issues. These results open new perspectives for the industrial use of nanoadditives in P-PVC matrices, identifying the possibility to use standard extrusion process to promote strong interactions with nanofillers. However, before technological applications, the influence of oxygen (by TGA under oxidative atmosphere) and weathering (ageing studies) effects must be investigated.

## Figures and Tables

**Table 1:** Composition of the Plasticized-Poly(vinyl chloride) (P-PVC) formulation.

Component	Amount (phr) [wt.%]
PVC K.70	100 [39.35%]
Coated CaCO3 (Atomfor S)	75 [29.52%]
Non stab.diisonoyl Phtalate	50 [19.68%]
Chloroparaffins (52%)	18 [7.08%]
Calcium Stearate	1.5 [0.59%]
Zinc Stearate	0.4 [0.16%]
Epoxidised soybeans oil	4 [1.57%]
Realube RL105	0.7 [0.28%]
Stearic Acid	0.3 [0.12%]
Irganox 1010	0.2 [0.08%]
Sb2O3	4 [1.57%]

**Table 2:** Formulations with a single nanoadditive.

Coding	Nanoadditive 1	phr [wt.%]
REF	-	-
IBuPOSSOH_0.62	Trisilanol heptaisobutyl POSS	-
PhPOSSOH_0.62	Trisilanol heptaphenyl POSS	0.62 [0.62%]
VyPOSS_0.62	Octvinyl POSS	0.62 [0.62%]
GlyPOSS_0.62	Glycidyl POSS	0.62 [0.62%]
X-ZEO_0.31	X-type zeolite	0.31 [0.31%]
X-ZEO_0.62	X-type zeolite	0.62 [0.62%]
HTLC_5	CO_3_-HTLC	5 [4.76%]

**Table 3:** Formulations with two nanoadditives.

Coding	Nanoadditive 1	phr [wt.%]	Nanoaditive 2	phr [wt.%]
REF	-	-	-	-
X-ZEO_0.31/GlyPOSS_0.62	X-type zeolite	0.31 [0.31%]	Glycidyl POSS	0.62 [0.62%]
X-ZEO_0.62/GlyPOSS_0.62	X-type zeolite	0.62 [0.62%]	Glycidyl POSS	0.62 [0.62%]
X-ZEO_1.25/GlyPOSS_0.62	X-type zeolite	1.25 [1.23%]	Glycidyl POSS	0.62 [0.62%]
HTLC_5/GlyPOSS_0.62	CO_3_-HTLC	5 [4.76%]	Glycidyl POSS	0.62 [0.62%]
HLTC_5/VyPOSS_0.62	CO_3_-HTLC	5 [4.76%]	Vinyl POSS	0.62 [0.62%]

**Table 4:** Standard methods.

Test	Standard
Tensile	ASTM D638
Sh. A (15”) hardness	ISO 868
HCl evolution	UNI EN ISO 182-3:2003 part 3
HCl evolution	CEI EN 50267-2-1/IEC 60754-1
Fire	UL94
LOI	ASTM D 2863

**Table 5:** HCl evolution according to UNI EN ISO 182-3:2003 test method for P-PVC and nanocomposite formulations, Limiting Oxygen Index (LOI) according to ASTM D 2863.

Coding	Thermal Stability [min]	Residual th. stab. [min] after 168 h @ 80 °C	LOI [% O_2_]
REF	40	-	24.5
X-ZEO_0.31	40	-	25.5
X-ZEO_0.62	45	-	25.5
X-ZEO_0.31/GlyPOSS_0.62	55	-	25.5
X-ZEO_0.62/GlyPOSS_0.62	55	-	25.0
IBuPOSSOH_0.62	45	-	25.0
PhPOSSOH_0.62	40	-	25.5
VyPOSS_0.62	40	-	25.0
GlyPOSS_0.62	60	35	25.5
HTLC_5/GlyPOSS_0.62	165	140	25.5
X-ZEO_1.25/GlyPOSS_0.62	40	35	25.0
HTLC_5/VyPOSS_0.62	165	140	25.5
HTLC_5	165	140	26.0

**Table 6:** HCl evolution according to CEI EN 50267-2-1/IEC 60754-1 method for P-PVC and nanocomposite formulations.

Coding	HCl Evolution [mg/g]	Variation [%]
REF	140	-
HTLC_5	125	−10.7
GlyPOSS_0.62	130	−7.1
HTLC_5/GlyPOSS_0.62	120	−14.3

## Supplementary Materials

The following are available online at https://www.mdpi.com/2073-4360/11/7/1105/s1, Figure S1: TGA analyses of reference P-PVC and X-type zeolite and X-type zeolite/GlyPOSS formulations in the compositional range 0.31–0.62 phr, in ramp heating 10 °C/min, Ar flow (20 mL/min) from RT to 800 °C (left) and isothermal measurements (Ar, 265 °C) (right), Table S1: torque value during extrusion with different PVC nanocomposite formulations; Table S2: Mechanical properties for tensile test and hardness test.
