# Supplementary material for: The Use of POSS-Based Nanoadditives for Cable-Grade PVC: Effects on Its Thermal Stability
Source: Polymers (Basel). 2019 Jun 29;11(7):1105. doi: 10.3390/polym11071105 (PMC6681030; doi:10.3390/polym11071105)
Supplement: Supplementary file 1 [file polymers-11-01105-s001.pdf]

Supplementary Information file of the paper

The use of POSS-based nanoadditives for Cable-grade PVC: effects on the thermal stability

Luca Palin[1,2], Giuseppe Rombolà[1,2], Marco Milanesio[1,2], Enrico Boccaleri*[1,2]

[1] Dipartimento di Scienze ed Innovazione Tecnologica (DiSIT), Università del Piemonte Orientale, Viale T. Michel, 11, 15121 Alessandria (I)

[2] Nova Res S.r.l., Via D. Bello, 3, 28100 Novara (I)

Correspondence should be addressed to Enrico Boccaleri; enrico.boccaleri@uniupo.it

Identification of commercial additives reported in Table 1.

Atomfor S is a calcium carbonate coated with stearic acid produced by Omya
Chloroparaffins are chlorinated paraffins containing 52% wt. of Cl named Cereclor and produced by Ineos
Epoxidized soybean oil (ESBO) is a plasticizer and a scavenger for hydrochloric acid produced by KH Chemicals
Jayflex™ DINP plasticizer is the largest-volume general-purpose high-molecular-weight plasticizer for PVC, produced by ExxonMobil Chemical
Calcium and Mg stearate and stearic acid are heat stabilisers and are produced by Baerlocher
Sb_2O_3 is a flame retardant produced by Biesterfeld Spezialchemie
Realube is a lubricant (basically a paraffinic wax) produced by Reagens
Irganox is a commercial antioxidant additive (based on hindered phenolic compounds) produced by BASF

Table S1: Torque value during extrusion with different PVC nanocomposite formulations.

Coding	Screw speed	Motor Torque
	rpm	%
REF	90	35
IBuPOSSOH_1.25	90	17
REF	150	24
IBuPOSSOH_1.25	148	20
PhPOSSOH_2.5	150	22
IBuPOSSOH_0.62	150	22
PhPOSSOH_0.62	151	22
VyPOSS_0.62	145	22
GlyPOSS_0.62	149	22
X-ZEO_0.31	153	24

X-ZEO_0.62	149	26
X-ZEO_0.31/GlyPOSS_0.62	152	20
X-ZEO_0.62/GlyPOSS_0.62	149	22
X-ZEO_1.25/GlyPOSS_0.62	150	25
HTLC_5	156	24
HTLC_5/GlyPOSS_0.62	150	23
HLTC_5/VyPOSS_0.62	151	24

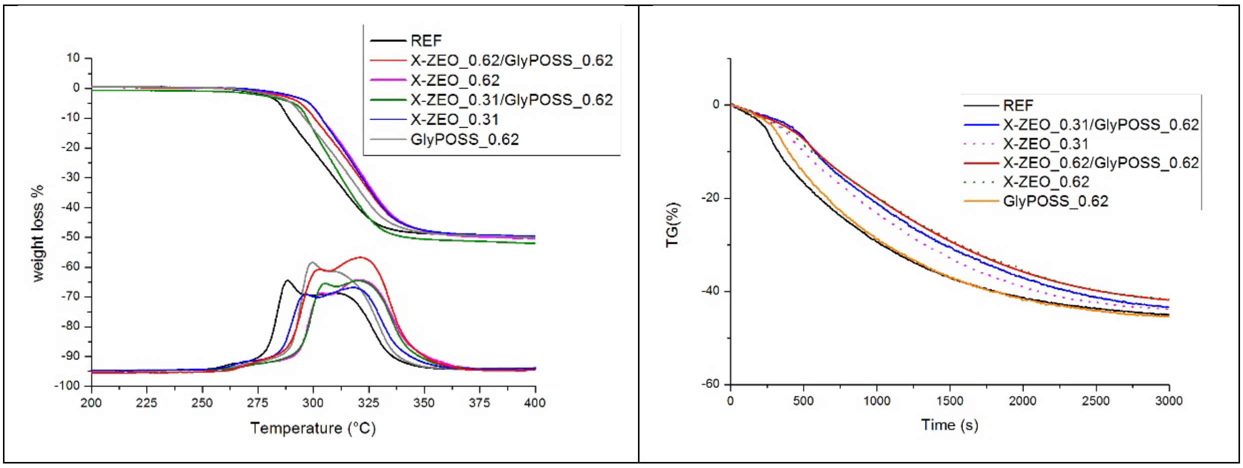

Figure S1: TGA analyses of reference P-PVC and X-type zeolite and X-type zeolite/GlyPOSS formulations in the compositional range 0.31–0.62 phr, in ramp heating 10°C/min, Ar flow (20 mL/min) from RT to 800°C (left) and isothermal measurements (Ar, 265°C) (right).

Table S2: Mechanical properties for tensile test and hardness test.

	Tensile Modulus (MPa)	Stress at break (MPa)	Strain at break (%)	Hardness Sh. A, 15′′
REF	10.00 ±0.33	13.8±0.13	320±3.5	80.0±1.6
X-ZEO_0.31	9.57±0.13	13.2±0.90	310±15	77.5±1.5
X-ZEO_0.62	9.69±0.56	13.2±1.26	310±12	77.5±1.5
X-ZEO_0.31/GlyPOSS_0.62	8.81±0.18	13.2±0.16	330±6.3	77.0±1.5
X-ZEO_0.62/GlyPOSS_0.62	8.44±0.27	12.4±0.77	310±6.8	78.0±1.5
IBuPOSSOH_0.62	8.93±0.13	13.6±0.32	320±2.1	77.5±1.5
PhPOSSOH_0.62	8.95±0.18	13.0±0.13	320±1.6	78.0±1.5

VyPOSSOH_0.62	8.91±0.14	13.0±0.01	310±1.4	81.0±1.6
GlyPOSS_0.62	8.87±0.10	13.5±0.32	320±6.6	79.5±1.6
HTLC_5/GlyPOSS_0.62	9.35±0.18	12.5±0.14	310±0.92	80.0±1.6
X-ZEO_1.25/GlyPOSS_0.62	9.12±0.01	12.3±0.05	300±2.3	77.0±1.5
HTLC_5/VyPOSS_0.62	9.44±0.01	12.7±0.01	310±0.0	81.5±1.7
